# Leisure-time physical activity and risk of depression: A dose-response meta-analysis of prospective cohort studies

**DOI:** 10.1097/MD.0000000000029917

**Published:** 2022-07-29

**Authors:** ZhiGuang Guo, Rui Li, Songtao Lu

**Affiliations:** a School of Sports Health, HuBei University of Chinese Medicine, Wuhan, China; b School of Physical Education, Central China Normal University, Wuhan, China; c Institute of Physical Education, Kashgar University, Kashi, China; d School of Sports, Wuhan University of Science and Technology, Wuhan, China.

**Keywords:** depression, dose-response, physical activity, meta-analysis

## Abstract

**Background:**

There has never been a dose-response meta-analysis of the relationship between physical activity and the risk of depression. Hence, we aimed to explore the dose-response relationship between leisure-time physical activity (LTPA) and the risk of depression through a meta-analysis to provide a basis for the prevention of depression.

**Methods:**

PubMed and Web of Science databases were searched using a computer to collect prospective cohort studies on the relationship between LTPA and depression between January 1997 and July 2021. A dose-response meta-analysis was performed using the Stata 14 software to calculate the combined effect size relative risk (RR and 95% confidence interval CI).

**Results:**

Twelve cohort studies included 310,359 subjects who met the inclusion criteria. The categorical dose-response analysis results showed that the risk of incident depression was 27%, 17%, and 8% lower for the light, moderate, and highest dose LTPA participants, respectively (RR: 0.73, 95% CI: 0.64–0.82; RR: 0.83, 95% CI: 0.78–0.87; RR: 0.92, 95% CI: 0.86–0.99), compared with the lowest LTPA category. Continuous dose-response analysis showed a nonlinear relationship between LTPA and the risk of incident depression (*P* = .04). The risk of incident depression was reduced by 3% (RR: 0.97, 95% CI: 0.95–0.98) for every 5 MET-h/week increase in LTPA < 25 MET-h/week; when LTPA was higher than 25 MET-h/week, a 4% increase in the risk of depression for every 5 MET-h/week increase was observed (RR: 1.04, 95% CI: 1.02–1.05).

**Conclusions:**

There was a nonlinear relationship between LTPA and the risk of incident depression. Moderate and low doses of LTPA were protective factors in preventing the risk of incident depression, while high doses of LTPA may increase the risk of incident depression.

## 1. Introduction

Depression affects approximately 300 million individuals worldwide.^[1]^ The treatment and prevention of depression have received particular focus from the global academic community, and it requires positive interventions, including modification of established risk factors. A potentially modifiable risk factor for depression is sedentary behavior or a low level of physical activity.^[2]^ Fifty percent of patients with severe depressive disorder cannot reach the recommended level of physical activity (150 min of moderate-intensity physical activity per week).^[3]^ Increasing physical activity is considered a possible protective factor against depression risk in many previous studies, and physical exercise can reduce depression symptoms in patients with potential depression.^[2–4]^ Therefore, the extent of physical activity to prevent depression, which involves the duration, intensity and frequency of physical activity, must be known.

There are several empirical studies on the dose of physical activity to prevent the risk of depression. For example, Fernandez-Montero et al considered that the total dose of sports (MET) and the duration of sports activities were significantly correlated with the incidence rate of depression; however, there was no correlation with the intensity of sports activities.^[5]^ Kim et al determined the best physical activity volume of 1200 to 3000 METs-min/week to reduce the onset of depressive symptoms in a cohort study. The “U” curve represents the dose-response relationship between physical activity and depression incidence rate.^[6]^ Kandola et al,^[7]^ a recent research paper published in Lancet, confirmed that increasing physical activity and reducing sedentary behavior in adolescents could reduce the incidence and prevalence of depression. These empirical studies have verified the relationship between various doses of physical activity and the risk of depression.

Relevant systematic reviews and meta-analyses have also confirmed that physical activity is a protective factor against depression.^[2,8]^ Even a small amount of physical activity (such as walking, 150 minutes per week) can reduce the incidence of future depression.^[2]^ A meta-analysis of cohort studies by Schuch et al concluded that physical activity in both age and geographic area could reduce the incidence rate of depression.^[9]^ These review studies further confirm the protective effect of physical activity on the risk of depression. However, no systematic review of the above empirical studies has been known to quantify the preventive effects of various doses of physical activity on depression. Therefore, in this study, we conducted a dose-response meta-analysis to quantify the relationship between different physical exercise doses and the risk of depression to bridge the current international guidelines for physical activity, which specifically aim to reduce the risk of depression. These major quantitative characteristics may have important clinical significance and practical guidance for exercise prescriptions to promote mental health.

## 2. Materials and Methods

### 2.1. Inclusion criteria

First, the inclusion criteria and each included article were discussed by 3 authors. The inclusion criteria were as follows: (1) the study was a published cohort study or prospective or retrospective observational study, and the baseline subjects were healthy people without a history of depression. (2) The categorical level of physical activity in the baseline survey population was ≥ 3, reflecting the dose-response trend. (3) The study should report the duration, frequency, or intensity of physical activity or MET dose that can be calculated into MET. (4) The relationship between the level of sports activity and the risk of depression (incidence rate rather than prevalence) is the relative risk (RR) and its 95% confidence interval (95% CI), or the original data can be used to calculate the above indicators. Repetitively published articles were excluded from the analysis. Two authors independently decided against inclusion, and the inconsistency was determined by group discussion. In this study, to highlight the role of physical activity or exercise, LTPA was termed physical activity. Light intensity physical activity, moderate-intensity physical activity, vigorous-intensity physical activity, and moderate-to-vigorous intensity physical activity are abbreviated as LPA, MPA, VPA, and MVPA, respectively.

### 2.2. Search strategy

We searched the literature on the relationship between sports activities and the incidence rate of depression from PubMed (from 1980 to date) and the Web of Science Database (from 1980 to date). The terms “exercise or physical activity or sport or walking or motor activity” and “depression” were used in the search strategy to screen whether they were cohort studies or prospective studies. The latest search date was May 2021, with no language restrictions. The reference lists of the selected articles and related reviews were screened step-by-step to identify potentially related studies.

### 2.3. Quality evaluation

The Newcastle Ottawa Scale (NOS) was used to evaluate the quality of the literature. This scale assesses the quality of the included cohort studies with scores ranging from 0 (indicating poor-quality studies) to 9 (indicating high-quality studies). The NOS contains 8 items, categorized into 3 dimensions: selection, comparability, and outcome (cohort studies). A star rating system from NOS is used to quantitatively estimate the quality of the included studies, which considers a total star of up to 9 and studies with >6 stars were evaluated as high quality. Hence, the scores of 0 to 3, 4 to 6, and 7 to 9 stars were evaluated as low, medium, and high quality, respectively.^[10]^ Each article was evaluated independently by 2 authors and cross-checked. If the quality evaluation of the literature was inconsistent, the group focused on obtaining the final quality score.

### 2.4. Statistical analysis

We performed a separate meta-analysis for both categorical and continuous variables to evaluate the association between LTPA and the risk of depression. Due to different LPTA doses of cutoff points in the included studies for categories, we performed relative risk estimates using the method recommended by Orsini et al.^[11]^ When LTPA was reported as a categorical variable, we compared the corresponding RR values between the highest and lowest LTPA categories. A categorical dose-response analysis was performed to generate 4 types of PA: lowest, light, moderate, and highest. The lowest and highest PA categories corresponded to the lowest and highest groups for each included study, respectively. For studies with at least 3 exposure categories, the second and third highest PA categories corresponded to the light and moderate groups, respectively. The combined RRs and 95% CIs associated with different PA categories were calculated by comparing each PA category (highest, moderate, and light PA) with the lowest PA categories using random-effects modeling techniques. Using stata14.0 software for meta-analysis, *P* values <.05 were considered statistically significant with all double-sided testing. The effect value-corrected risk ratio (RR) and 95% confidence interval (CI) of each study’s highest dose LTPA group were combined. A random-effects model was used to connect the effect values. I^2^ statistics were used to evaluate and describe the percentage of variation in the study.^[12]^ We assessed the potential for publication bias using Egger linear regression test and Begg rank correlation test. As for sensitivity analysis, we checked whether the combined effect of the remaining studies had changed by excluding 1 study at a time. A subgroup meta-analysis was performed by classifying the intensity of LTPA, sex, age, area of study subjects, and quality of the study. Simultaneously, the heterogeneity of each subgroup was examined using meta-regression.

When LTPA was reported as a continuous variable, a random-effects model was used to examine the reduction in the risk of depression due to LTPA. The median or mean LTPA levels within the exposure categories were assigned to the corresponding RRs. If these results were unreported, we used the midpoint between the lower and upper boundaries of the exposure category and generally assumed the interval in the highest category to be equivalent to that in the next highest exposure category. We used MET-h/week values as the compendium of physical activities.^[13]^ Generally, a value of 3 MET is used for light physical activities or walking, 4.5 MET for moderate LTPA, 5 MET for moderate-to-vigorous LTPA, and 8 MET for vigorous LTPA/sports/running.

We calculated study-specific slopes (linear trends), 95% CIs from the natural logs of the reported RRs and CIs across categories of LTPA measures, using the robust error meta-regression method described by Xu et al.^[14]^ This method was based on a 1-stage approach that treats each study as a cluster of the whole sample and considers the within-study correlations by clustered robust error. Known cardiorespiratory fitness and RRs with variance estimates for at least 2 quantitative exposure categories were required. Based on the goodness of fit test of the model, and the model parameters of the invalid hypothesis test, the Stata software XBLC command was used to draw a dose-response curve.^[15]^

## 3. Results

### 3.1. Literature search and study characteristics

**Figure 1.**
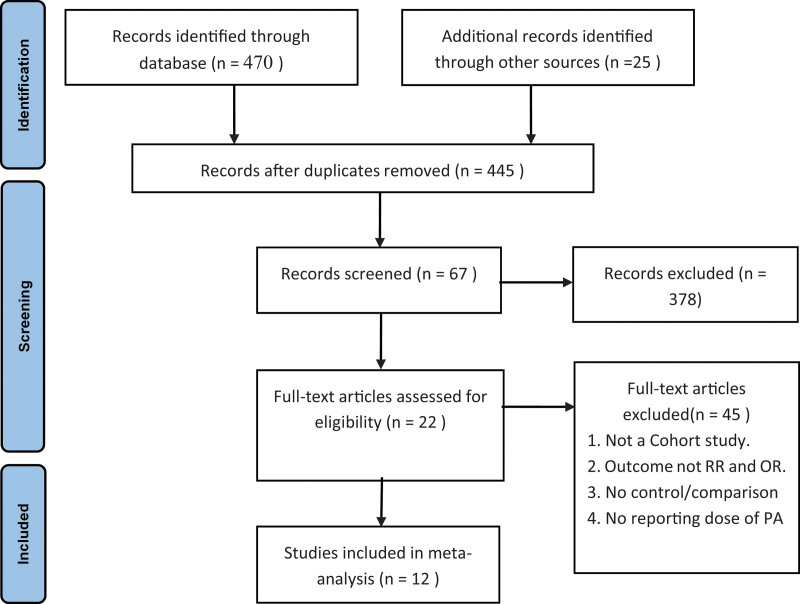
Flowchart of the literature search and literature inclusion.

According to the inclusion and exclusion criteria, 14 groups of 12 cohort studies were included with 570,244 subjects.^[5,6,10,16–24]^ The steps of retrieval and inclusion steps are illustrated in Figure 1. The characteristics of the included studies are presented in Table 1. According to the NOS score, 6 works of literature were ≥7 points, which was high-quality literature, and the others were medium quality literature. Among the 12 studies, 5 were from America,^[10,16,20,22,23]^ 2 were from Asia,^[6,21]^ 4 were from Europe,^[5,17,18,24]^ and one was from Oceania.^[19]^

**Table 1:** Study characteristics.

Author (year, country)	Study name	Participants, women, %	Mean age, y	Follow-up, y %	Depression evaluation	PA evaluation tool	Quality score
Chang et al^[22]^; USA	TLSA	21,728 (100)	≥65	10 (90%)	CES-D	Self-report PA	9
Fernandez et al^[5]^; ES	SUN	15,488 (60)	37 ± 12	10.5 (69%)	DSM-IV	LTPA^[25]^	8
Gallegos et al^[23]^; MX	HWCS	1047 (77.5)	Adults	6 (NA)	CES-D	LTPA^[25]^	8
Kim et al^[6]^; KR	KSHS	107,901 (32.9)	18–64	2.2 (NA)	CES-D	IPAQ^[26]^	8
Kuwahara et al^[21]^; JP	JECOH	29,082 (15.1)	20–64	4.7 (NA)	CES-D	Self-report PA	7
Lucas et al^[20]^; USA	TLSA	49,821 (100)	60–70	10 (NA)	CES-D	Self-report PA	7
Pavey et al^[19]^; AU	ALSWH	9091 (100)	22–27	12 (NA)	CES-D	IPAQ^[27]^	6
Sánchez-Villegas et al^[18]^; ES	SUN	11,800 (58)	26–50	8.5; 90%	DSM-IV	LTPA^[25]^	7
Smith et al^[16]^; USA	THHP	3741 (0)	71–93	8 (40.5)	CES-D	Self-report PA	7
Slykerman et al^[24]^; NZL	ABC	491 (50.7)	11	12 (56.4%)	CES-D	AM71256 Accelerometer	6
Wise et al^[10]^; USA	TBWHS	59,000 (100)	21–69	4; 80%	CES-D	PA^[28]^	5
van Gool et al^[17]^; NL	TLMAS	1169 (47.6)	24–81	6; 62.8%	CES-D	Self-report PA	7

### 3.2. Categorical dose-response association between physical activity and depression risk

**Figure 2.**
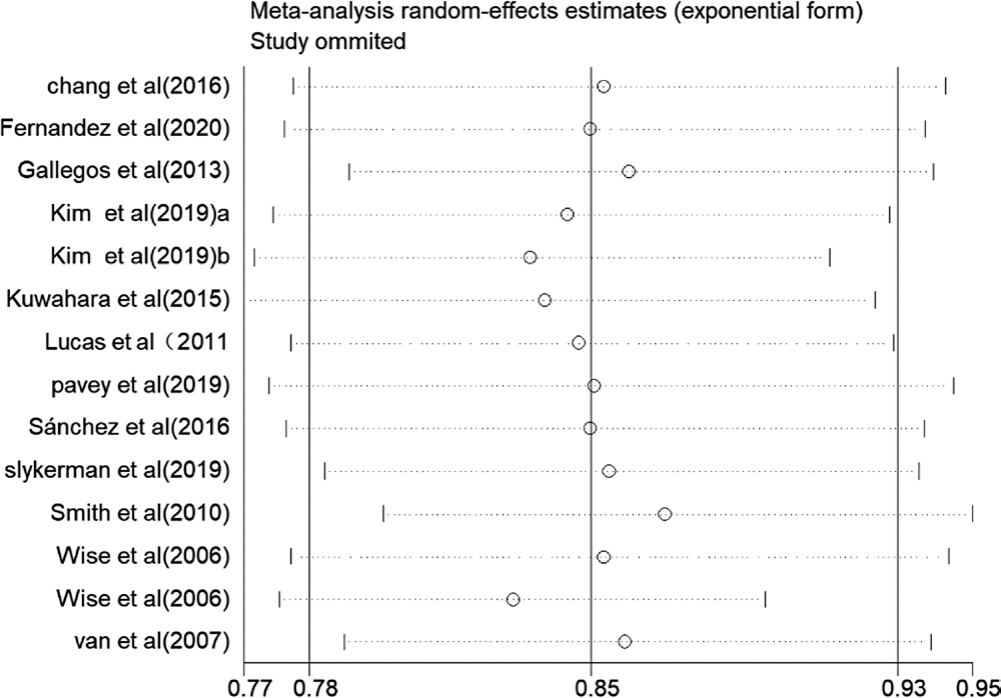
Sensitivity analysis.

**Figure 3.**
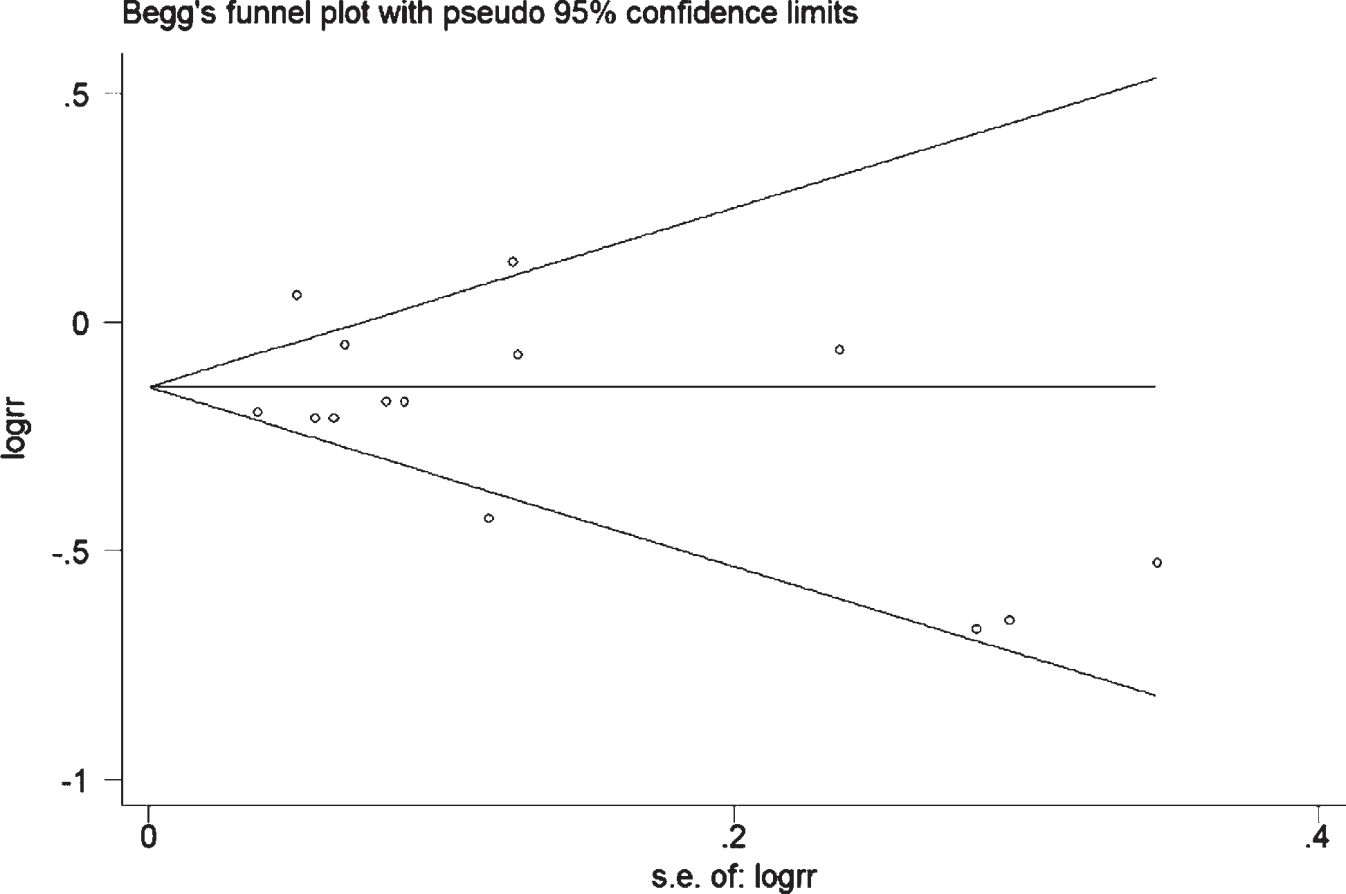
Publication bias analysis.

Table 2 presents the pooled estimates of RR for incident depression associated with different categories of LTPA and the subgroup analysis. For all LTPA categories, participants had a 23% lower risk of incident depression than the lowest PA group (pooled RR, 95% CI: 0.77 [0.68–0.86]; I^2^ = 69%). One-by-one exclusion sensitivity analysis showed no significant change in the total effect value (as presented in Fig. 2), and our study did not show considerable publication bias (as presented in Fig. 3). The risk of incident depression was 27% lower among the participants in the light PA category than in the lowest-level LTPA category (pooled RR, 95% CI: 0.73 [0.64–0.82]; I^2^ = 43%). Moderate and highest LTPA category participants also had a 17% and 8% lower risk of incident depression compared with the lowest PA group (RR, 95% CI: 0.83 [0.78–0.87], I^2^ = 46%; RR, 95% CI: 0.92 [0.86–0.99]; I^2^ = 79%).

**Table 2:** Main result and subgroup analysis

Subgroup (study n)	RR	I^2^	P^1^	P^2^
Total LTPA (14)	0.77 (0.68–0.86)	69%	.45	–
Categorical dose				.027
Light	0.732 (0.64–0.82)	43%	.135	
Moderate	0.831 (0.78–0.87)	46%	.110	
Highest	0.929 (0.86–0.99)	79%	.002	
Intensity				.62
LPA (4)	0.80 (0.50–1.09)	88%	<.01	
MVPA (3)	0.80 (0.739–0.87)	0%	.61	
LPA and MVPA (7)	0.86 (0.77–0.95)	77.5%	.04	
Sex				.26
Male (2)	0.77 (050–1.05)	69%	.046	
Female (7)	0.90 (0.80–0.99)	77%	.001	
Mix (5)	0.77 (0.64–0.90)	61%	.023	
Age				.81
Teenagers (2)	0.81 (0.70–0.90)	7%	.30	
Adult (6)	0.88 (0.81–0.96)	0%	.4	
Elderly (3)	076 (0.62–0.89)	44%	.164	
Mix (6)	0.85 (0.68–1.02)	82%	<.01	
Depression measurement				.241
CES-D (12)	0.85 (0.81–0.88)	74	<.01	
DSM (2)	0.84 (0.74–0.94)	0	1.0	
Quality of study				.72
>6	0.82 (072–0.91)	61%	<.01	
«6	0.86 (0.73–1.00)	83%	<.01	

Meta-regression analysis was used to test the sources of heterogeneity (Table 2). It was observed that only the *P* value of heterogeneity in the different dose subgroup categories was 0.02 with significant heterogeneity in each study according to the subgroup. Therefore, different doses of physical activity have different effects on preventing depression. In addition, the heterogeneity analysis within subgroups showed that the heterogeneity was significant in the group with the highest dose (>50 MET/week), which may indicate that the higher the dose of physical activity, the more uncertain about the preventive effect on depression. Further analysis showed significant heterogeneity in the LPA low-intensity, female, mixed-age, Center for Epidemiological Studies Depression evaluation method, and study quality subgroups, which may still be due to different doses of physical activity. In addition, these subgroups may also be important influencing factors that need to be further examined in future studies.

### 3.3. Continuous dose-response association between physical activity and depression risk

**Figure 4.**
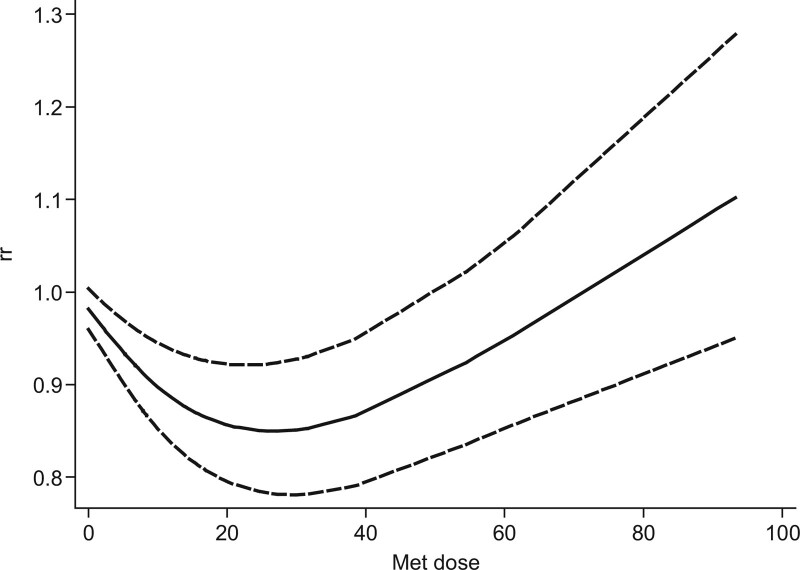
Continuous dose-response relationship analysis.

Figure 4 presents the continuous dose-response association between quantitative estimates of PA (MET-min/week) and depression risk. These pooled results showed a consistent, inverse dose-response association between PA and the risk of depression. The nonlinear shape was similar to that of the U-shaped dose-response curve (p_non-linearity_ = 0004 < .05, I^2^ = 16.56%).

According to the nonlinear results, when physical activity was <25 MET-h/week, the RR of depression risk was reduced by 3% (RR: 0.97 [0.95–0.98]) for every 5 MET-h/week increase; when physical activity was >25 MET-h/week, the increased physical activity did not further reduce the risk of depression, that is, the risk of depression increased by 4% for every 5 MET-h/week increase (RR: 1.04 [1.02–1.05]).

## 4. Discussion

To the best of our knowledge, this is the first dose-response association analysis of LTPA and the risk of depression. By integrating the results of the cohort study, compared with the lowest LTPA category, we determined the risk ratio of the different category doses of physical activity to depression risk was 0.77, 0.73, 0.83, 0.92 for the total, light, moderate, highest level dose, which was more specific than the meta-analysis results of Schuch et al (RR for the total level: 0.83)^[3]^ and Dishman RK et al (OR for the total level: 0.79).^[29]^ As Zhang et al described in the conventional meta-analysis method, only the data of multiple groups between the highest and lowest doses were selected for comparison without a specific dose level, such that the data were inconsistent.^[10]^ This treatment greatly enhanced the accuracy of the combined effect value of this dose-response meta-analysis.

The continuous dose-response relationship showed that when the level of LPTA > 25 MET-h/week, the risk of depression increased, indicating a threshold effect. The practical significance of this conclusion may be that approximately 25 MET-h/week is the best dose for a protective effect against depression. The significance of increasing the benefit of 5 MET-h/week is that another hour of MVPA or 2 hours more of LPA once a week will decrease the risk of depression by 3%. If the total dose is more than 25 MET-h/week, the incidence rate of depression may increase by 4% with another hour of MVPA or 2 hours more of LPA once a week. This study’s conclusion is similar to that of Kim et al research with 20 to 50 MET-h/week for the dose range of the protective effect of depression, and we confirm that the dose-response relationship is a nonlinear U-shaped one.^[6]^ We also identified high-intense PA or exercise as factors that increase the risk of depression, which enriches the conclusions of previous meta-analyses of the association between physical activity and depression. Most of these studies merely demonstrated that physical-overall activity reduced the risk of depression.^[29–36]^ In contrast, our study sorted out the relationship between different physical activity levels and depression risk by categorical and continuous dose-response analysis. In addition, physical activity < 25 MET-h/week reducing the risk of depression still corresponds to 10 MET-h/week for the lowest dose and 20 MET-h/week for additional health benefits recommended by WTO.^[37]^

Heterogeneity is the most important index for evaluating the stability of meta-analysis results. However, the results of this study had a large heterogeneity in the combined effect value of all category doses, with heterogeneity I^2^ of 68% (14 studies). Through meta-regression analysis, it was found that the heterogeneity resulted from the different dose levels of LPTA on the effect value RR for incident depression. For example, in the included studies by Kim et al and Wise et al, the RR values were 1.14 and 1.06.^[6,10]^ however the RR values of Gallegos et al and Van et al were 0.51 and 0.52.^[17,23]^ These data appear to contradict our conclusions. In addition, Kim et al and Gallegos et al reported 97.1 and 20.8 MET-h/week respectively for the highest dose, which was the obvious difference when comparing almost the same lowest dose.^[6,10]^ Simultaneously, this study’s continuous nonlinear U-shaped dose-response relationship is more convincing explaining the heterogeneity. Compared to the heterogeneity I^2^ value of the study by Schuch et al, it was 0% (36 studies) with significant publication bias,^[30]^ whereas our study did not show any significant publication bias.

The results of this study are based on a large sample of a cohort study and the advantages of a multiyear follow-up survey; therefore, the results are relatively stable. Nevertheless, the following limitations may be found in this study. First, the literature we have included may be insufficient. A possible reason for this is that we set strict inclusion criteria. We needed the data reported to calculate the MET dose. Furthermore, the study data included at least 3 categories to set at least 2 nodes in the restricted cubic bar to generate 2 regression splines to judge whether the overall study was linear or nonlinear according to the second regression spline. Second, the methods of PA evaluation in this study were mostly subjective measurements, which may have led to inaccurate doses. We assumed that there had been no change in exercise or exercise habits for a long time in all cohort studies as in previous methods of dose-response meta-analysis related to physical activity topics,^[38–40]^ which will lead to inaccurate results. In addition, another limitation of these analyses is that included studies used different measures of physical activity and depression. Meanwhile, there were relatively limited data at higher physical activity doses, which may be the factors affecting robust conclusions. Finally, most cohort studies have not adjusted confounding factors. These studies have been based on the relative risk after exposure and nonexposure comparisons, so the conclusions of the cohort studies need to be interpreted with caution. These limitations should be addressed in future research.

## 5. Conclusions

There is a nonlinear dose-response association with a U-shape between LTPA and the risk of incident depression. Compared to the lowest LTPA category, the risk of incident depression was 27% lower among participants in the light PA category. The moderate and highest LTPA category participants also had a 17% and 8% lower risk of incident depression, respectively, compared with the lowest PA group. The risk of incident depression was reduced by 3% or increased by 4% by increasing another 5 MET-h/week when LTPA was lower or higher than 25 MET-h/week, respectively. High-intense PA or exercise may be a factor in increasing the risk of depression.

## Acknowledgments

We thank all the reviewers for their helpful comments.

## Author contributions

Conceptualization: Songtao Lu, Rui Li, and Zhiguang Guo,

Data curation: Songtao Lu, Rui Li,Zhiguang Guo

Formal analysis: Songtao Lu, Zhiguang Guo

Investigation: Songtao Lu, Zhiguang Guo

Methodology: Songtao Lu, Rui Li, Zhiguang Guo

Resources: Songtao Lu, Rui Li, Zhiguang Guo

Software: Rui Li, Zhiguang Guo

Supervision: Songtao Lu

Validation: Rui Li, Zhiguang Guo

Writing—original draft: Songtao Lu,Zhiguang Guo

Writing—review and editing: Songtao Lu
